# Infused polymers for cell sheet release

**DOI:** 10.1038/srep26109

**Published:** 2016-05-18

**Authors:** Nidhi Juthani, Caitlin Howell, Haylea Ledoux, Irini Sotiri, Susan Kelso, Yevgen Kovalenko, Amanda Tajik, Thy L. Vu, Jennifer J. Lin, Amy Sutton, Joanna Aizenberg

**Affiliations:** 1 Wyss Institute for Biologically Inspired Engineering, 60 Oxford Street, Cambridge, Massachusetts 02138, United States; 2 John A. Paulson School of Engineering and Applied Sciences, Harvard University, 29 Oxford Street, Cambridge, Massachusetts 02138, United States; 3 Department of Chemical and Biological Engineering, University of Maine, 5737 Jenness Hall, Orono, ME 04469, United States; 4 Department of Chemistry and Chemical Biology, Harvard University, 12 Oxford Street, Cambridge, Massachusetts 02138, United States; 5 Kavli Institute for Bionano Science and Technology, Harvard University, 12 Oxford Street, Cambridge, Massachusetts 02138, United States

## Abstract

Tissue engineering using whole, intact cell sheets has shown promise in many cell-based therapies. However, current systems for the growth and release of these sheets can be expensive to purchase or difficult to fabricate, hindering their widespread use. Here, we describe a new approach to cell sheet release surfaces based on silicone oil-infused polydimethylsiloxane. By coating the surfaces with a layer of fibronectin (FN), we were able to grow mesenchymal stem cells to densities comparable to those of tissue culture polystyrene controls (TCPS). Simple introduction of oil underneath an edge of the sheet caused it to separate from the substrate. Characterization of sheets post-transfer showed that they retain their FN layer and morphology, remain highly viable, and are able to grow and proliferate normally after transfer. We expect that this method of cell sheet growth and detachment may be useful for low-cost, flexible, and customizable production of cellular layers for tissue engineering.

Cell sheet engineering is a powerful and versatile technology with promise in a variety of applications^1^. Sheets of mesenchymal stem cells (MSCs), in particular, have been successfully used for multiple applications, including the repair of scarred myocardium after myocardial infarction^2^, the rebuilding of cartilage^3^, and the enhancement of healing in critical-sized femoral defects^4^. Although MSCs are among the most popular cell types for cell-based therapies due to their multilineage differentiation potential into osteogenic, chondrogenic, or adipogenic cells^5^, cell sheet engineering with other types of cells, including hepatocytes^6^, epithelial cells^7^, and myocardial cells^8^, has also demonstrated the power and versatility of this technology.

Currently, the primary commercially available mode of creating free-standing cell sheets is the use of surfaces coated with the thermoresponsive polymer poly(N-isopropylacrylamide) (pNIPAAm)^9^,^10^,^11^. The surface-bound pNIPAAm undergoes a reversible transformation from hydrophobic to hydrophilic upon lowering the temperature below 32 °C, at which point any cells that have been cultured on its surface begin to detach^12^,^13^. Although this temperature-responsive cell sheet release has proven to be effective across a wide range of applications, it is by nature subject to several limitations. First, the time to detach a cell sheet from the current commercially-available thermo-responsive cell sheet surfaces can be 40 min or more^14^, making it incompatible with high-throughput applications. Second, the need for temperature changes to release the cells from the surface may change the gene expression or cell function in some more sensitive cell lines^15^. Finally, creating pNIPAAm-coated surfaces for intact cell-sheet release requires electron-beam or vapor-phase polymerization equipment and facilities^16^, which are not very common in biological labs. While pre-coated thermo-responsive surfaces are commercially available (e.g., UpCell), these materials can be prohibitively expensive in the quantities necessary to optimize cell-sheet release with a new cell line or for a new application. Other stimuli-responsive surfaces for growing and detaching cell sheets have also been explored, including electro-responsive^17^ and photo-responsive materials^18^. Although improving in the areas of temperature changes and detachment time, these approaches also require highly specialized facilities, materials, or expertise to manufacture.

At the core of each of these cell-sheet detachment methodologies is the concept that cells growing on the surface need to be able to form strong attachments to each other, as well as attachments to the surface that are sufficiently strong, to allow normal growth and proliferation. Furthermore, at the desired time of sheet release, the surface should switch from sticky to non-sticky, reducing cell sheet/substrate attachment strength and thus facilitating the lift-off of an uncompromised cell sheet construct. Nature has already presented a way to create such a reversibly slippery surface in the form of the peristome of the *Nepenthes* pitcher plant. Under dry conditions, ants and other insects can walk over the peristome without difficulty. However, when it rains, a thin layer of water becomes immobilized on this surface, rendering it extremely slippery and causing any insects that attempt to cross it to fall into the plant’s cup for digestion^19^. Recently, our group introduced Slippery Liquid-Infused Porous Surfaces (SLIPS) as omniphobic, non-adhesive coatings based on this concept and demonstrated that they can be used to effectively repel everything from ice^20^ to blood^21^ to bacteria^22^ to crude oil^23^.

The simplicity of the immobilized liquid overlayer concept combined with its success has generated widespread attention in both the medical^24^ and industrial fields^25^, and given rise to new ways of immobilizing liquids on surfaces. One such method is the use of oil-infused polymers. In these systems, bulk polymeric materials such as fluorogels^26^ or polydimethylsiloxanes (PDMS)^27^,^28^ are exposed to an excess amount of a chemically-matched oil. The polymers absorb the oil, leaving a thin liquid layer on the material surface and holding a reservoir of the oil in the polymer bulk, thus allowing the reservoir oil to diffuse to the interface and replenish the surface liquid layer as it becomes depleted. These materials have proven highly effective at resisting bacterial adhesion under both static and flow conditions^27^,^28^. We anticipated that poor adhesion to these types of surfaces could also offer opportunities in areas other than antifouling materials. In one example of this, we show here how slippery surfaces made using oil-infused polymers can be used in tissue engineering for the growth and detachment of mesenchymal stem cell sheets.

## Results

To prepare the oil-infused surfaces for cell growth, we first coated the bottom of plasma-treated 6-well TCPS plates with 1.08 g (±0.05 g) of uncured polydimethylsiloxane (PDMS) polymer, resulting in a 1.2 mm-thick coating covering the 35 mm well after curing. Trimethoxy-terminated silicone oil (10 cSt, ~2 mL) was then introduced on top of the PDMS coating and allowed to infiltrate the polymer for 48 h to ensure full uptake (Supplementary Fig. 1). On average, the polymer coatings took up 0.49 mL (±0.02 mL) of the oil, resulting in a swelling ratio of 1.42 (±0.01) by mass. Measurement of the elastic modulus of the infused polymer was performed via nanoindentation. At the 10:1 base to crosslinker ratio used for cell growth, a slight decrease from 2.12 (±0.09) to 1.47 (±0.05) MPa was observed (Supplementary Fig. 2). No delamination of the infused PDMS from the TCPS well surface occurred. Before cells were introduced to the system, excess surface oil was removed by washing the surfaces with water, blotting with a silicone sponge, and finally washing with ethanol.

**Figure 1:**
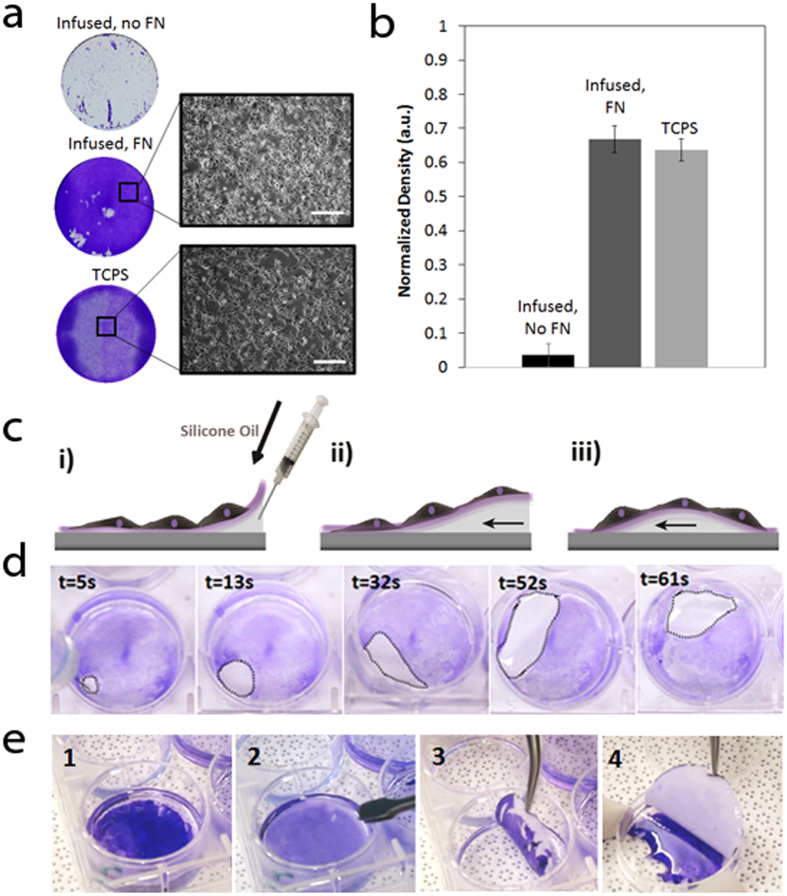
Growth and removal of cell sheets on oil-infused polymer surfaces. (**a**) Representative images of the cells grown in 35 mm diameter wells, stained with crystal violet (CV) for easier visualization (left), and light microscopy images of the cells (right; scale bars,100 μm). (**b**) Normalized density of cells grown on infused PDMS and the two control surfaces: infused PDMS without FN and unmodified TCPS. (**c**) Schematic of peeling of the cell sheet off the surface: (i)–(ii) silicone oil is added underneath the cells onto the surface of the infused PDMS, in order to allow the oil to penetrate between the surface of the infused PDMS and FN/cellular layer and create a small pool. (iii) The pool of silicone oil is moved around the well, delaminating the cell sheet from the surface as it travels. (**d**) Time lapse images of a pool of silicone oil (highlighted by a dotted line) moving around the well and delaminating the sheet from the surface. (**e**) Time-lapse images showing complete, intact cell sheet transfer from the 6-well plate to a petri dish. The cells are stained with crystal violet for easier visualization. 1) The intact cell sheet is first completely delaminated from liquid-infused polymer substrate. 2) A piece of filter paper is placed onto cell sheet. 3) The filter paper with the attached cell sheet is removed from the well. 4) The cell sheet is placed in a petri dish, and the filter paper is removed.

To create a more favorable environment for growth and proliferation, we coated our surfaces with human plasma fibronectin (FN) prior to cell seeding. We found the optimal concentration of FN for deposition to be at 1.25 μg/mL or approximately 0.52 μg/cm^2^ of infused polymer surface (Supplementary Fig. 3). Cell growth on FN-coated and uncoated infused PDMS was compared to unmodified TCPS controls. FN-coated infused PDMS showed a ~20-fold increase in cell proliferation based on the cell sheet density compared to uncoated surfaces (Fig. 1a,b). There was no significant difference (P = 0.907) between the densities of cells grown on fibronectin-coated infused and those grown on the TCPS control surface (Fig. 1b).

Once the cell sheet had grown to confluence or near-confluence, the cells were loosened by injecting an excess volume (approximately 850 μl total or 90 μl/cm^2^) of silicone oil between the FN layer/cell sheet and the infused surface. The newly-created oil pool was then gently maneuvered around the well by tilting the plate to separate the cell sheet from the surface (Fig. 1c,d). Using this simple procedure, an intact cell sheet could be released within minutes (5 ± 1.9 min on average) (Supplementary Video 1). As a control, this removal method was also attempted with cells grown on non-infused, FN-coated PDMS, as well as partially-infused PDMS (infused for only 4 h). It was found that in these cases the cell sheet could not be detached using added silicone oil, even when encouraging detachment with tweezers or a cell scraper, and simply shredded upon attempted removal. After the release from the FN-coated infused PDMS substrate, the cell sheet could be easily transferred to a new culture surface, for example using filter paper (Fig. 1e). A filter paper circle was gently placed in the well over top of the cell sheet. Media was aspirated from above the filter paper so that the cell sheet adhered to the filter paper through capillary force. The filter paper was then peeled off and transferred to the new culture surface where fresh media was added to detach the cell sheet (Supplementary Video 2).

**Figure 2:**
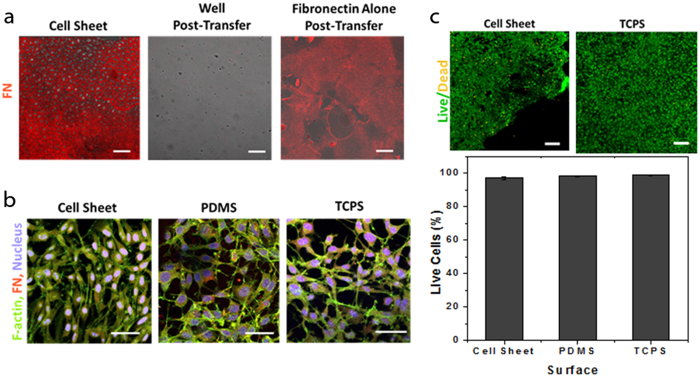
Characterization of the cell sheet post-transfer. **(a)** Confocal images of a transferred cell sheet 2 h after transfer stained for FN at the level of the cell-surface interface. There is a large amount of FN present, indicating the transfer of the ECM with the cell sheet. Images of the well post-transfer show little to no remaining FN after the transfer of the sheet. A FN layer with no cells was also transferred from the infused PDMS, suggesting that the removal mechanism is dominated by the protein/oil interaction rather than by cellular metabolism. Scale bar, 200 μm. (**b**) Confocal images of a transferred cell sheet stained for f-actin (green), fibronectin (FN, red) and nuclei (blue) at the level of the cells. Cells grown on TCPS and uninfused PDMS with FN shown for comparison. The morphology and arrangement of the cells appears similar between the sheet and the controls. Scale bar, 50 μm. (**c**) Confocal images of a transferred cell sheet and controls stained for live and dead cells. The percent live cells in the transferred sheet was found to be similar to the values calculated for cell sheets grown on untreated PDMS and TCPS. Scale bar, 200 μm.

Previous work on infused PDMS and other substrates for immobilized liquid layers has established that proteins and microorganisms are easily removed from these surfaces due to the inherent mobility of the liquid itself 21,22,23,27,28,29. To confirm that there was still an oil layer present after removing the excess oil before FN deposition, we visualized the surfaces over time immediately following oil removal. The results showed a replenishment of the surface oil over 48 h, supporting the hypothesis that the FN/cellular layer was sitting on top of an immobilized liquid layer when it was detached from the growth surface (Supplementary Fig. 4). The more direct evidence for this mechanism is the removal of the fibronectin extracellular matrix (ECM) layer together with the cell sheet. Images of the cell sheets 2 h after transfer showed a continuous FN layer underneath the cell sheet (Fig. 2a, left), while images of the well from which the sheet was transferred showed little to no FN remaining (Fig. 2a, middle). Even in the absence of the cells, the FN layer alone is successfully delaminated and transferred from the substrate (Fig. 2a, right), in full agreement with the concept that the mechanism of removal is dominated by the mobility of the liquid overlayer on top of which the adsorbed organic or biological material rests, as observed in previous investigations of immobilized liquid layers as anti-fouling surfaces. In addition, no difference in release time was observed for glutaraldehyde-fixed cells versus living cells.

Sheets were also immunostained for fibronectin, f-actin, and nuclei to visualize their cellular organization and structure compared to controls. The morphology of the cell sheets did not appear to change after removal from the PDMS surface (Fig. 2b), and continued to closely mimic that of a confluent cell layer on TCPS. Further imaging of stained sheets showed no change in morphology before and after transfer (Supplementary Fig. 5). Cell sheet viability 1 h after transfer was determined using a live/dead staining assay. Cell sheets grown on FN-coated PDMS and untreated TCPS were also stained as positive controls. Visually, there was no difference observed between the sheet after transfer and the TCPS controls. Quantitatively, the cell sheet showed 97.2% (±0.8%) live cells, compared to 98.3% (±0.3%) and 98.7% (±0.5%) for the cell sheets grown on fibronectin-coated PDMS and TCPS, respectively (Fig. 2a).

**Figure 3:**
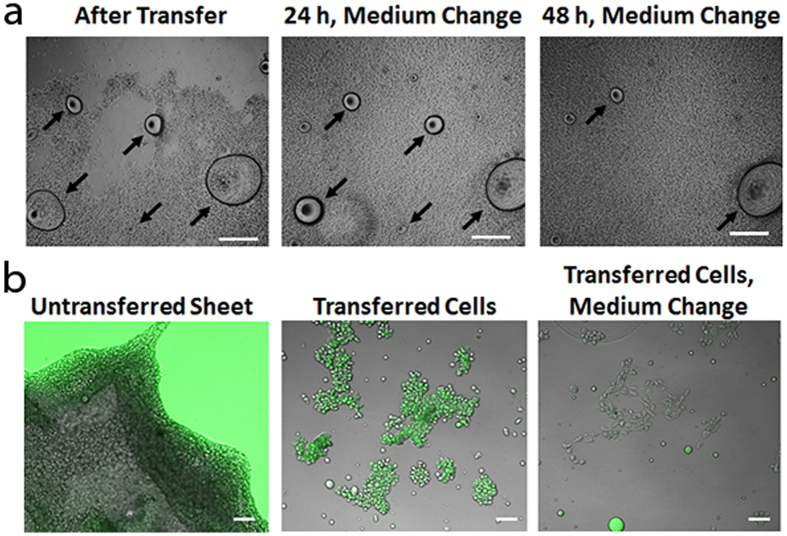
Cell Sheet Proliferation Post-Transfer. (**a**) Light microscopy images of the same region of a cell sheet grown on infused PDMS and transferred to TCPS immediately after transfer, 24 h after transfer with a change of medium, and after 48 h with another change of medium. The silicone oil drops (indicated by arrows) that are present are progressively washed away, and the sheet shows normal growth and proliferation. Scale bar, 1 mm. (**b**) A cell sheet that has been loosened, but not transferred, using dyed silicone oil (left image). The dyed oil has coated the substrate and the edges of the cell sheet. Small groups of transferred cells show a coating of the dyed oil (center image), however the dye is washed away with a change of the medium (right image). Scale bar, 200 μm.

In order to confirm that the cells within the sheet could continue to grow, we monitored the sheets for up to 5 days after transfer onto fresh unmodified TCPS surfaces. Normal growth and proliferation was observed: Fig. 3a shows a transferred cell sheet with a large gap immediately after transfer, after 24 h and a change of medium, and after 48 h and another change of the medium. The cells of the sheet have grown completely over the entire surface area after just 24 h. However, we observed that in using this method small oil droplets were sometimes present on the sheets immediately after transfer. These droplets could be washed away through normal changes of growth medium and did not appear to disrupt cell growth.

To further investigate the localization of the excess silicone oil on the cell sheet after transfer, we used a silicone oil-soluble fluorescent dye to perform the cell release (Fig. 3b). The leftmost image of Fig. 3b shows that the dyed oil has indeed spread evenly across the surface and appears to be present on the edges of the sheet. The center image shows groups of cells that were transferred, and are coated with the dyed oil. However, after a change of the medium, the amount of dyed oil on the cell surface decreases significantly (rightmost image).

## Discussion

In summary, we have demonstrated how oil-infused polymers can be used as cell sheet release surfaces for MSCs. A thin layer of PDMS was used to coat the bottom of a TCPS plate, then infused with silicone oil to produce the substrate. After treatment to remove excess silicone oil, the surface of the infused layer was coated with fibronectin to encourage cell growth and proliferation. Mouse MSCs were then seeded on the surface. After the cells had grown to confluency or near-confluency, the sheet was released from the surface by introducing an excess amount of silicone oil in a pool underneath the cell sheet by way of a syringe. The excess oil was able to slide underneath the cell sheet, releasing it from the surface. The released cell sheet could then be transferred to a new surface by means of a sheet of filter paper. Immunostaining of the cell sheets post-transfer showed no significant differences in the amount of fibronectin or f-actin per nucleus compared to cells grown on tissue culture polystyrene.

Over the last few years, a growing body of literature has emerged on the use of immobilized liquid overlayers, such as the ones used here, to repel a range of materials including bacteria and various biomolecules. The concept is that organic, inorganic, or biological substances can settle on the liquid layer, but are easily removed by any disturbance to the system due to the liquid nature of the surface they sit on^22^,^23^,^27^,^28^. We hypothesized and have demonstrated here that such a phenomenon can be used not only for the repulsion of unwanted material in various antifouling applications, but can be highly beneficial in tissue engineering for cell culturing purposes. We have shown that the mechanism, by which our cell sheet release system functions, is fundamentally no different from that described for the fouling-release capability of slippery surfaces; that is, the presence of the liquid overlayer supports cell culture and provides an easy transfer of the grown cell sheets due to their negligible adhesion to the underlying substrate. To achieve this, we first coat the slippery surface with an FN layer that denatures and forms a web on the surface of the liquid layer, which is initially thinned by an oil-removal step immediately prior to the FN deposition. The thinness of the layer, as evidenced in Supplementary Fig. 4 (t = 0), limits the mobility of any liquid molecules on the surface and allows the deposited FN layer to be stable enough for initial cell attachment and proliferation. Over time, the oil overlayer gradually thickens due to the diffusion of the oil from the polymer bulk (Supplementary Fig. 4), providing more mobility to the surface liquid film that supports the FN layer and the increasingly dense cellular sheet.

To fully release the cell sheet construct from the substrate, an excess volume of silicone oil is introduced in between the surface of the infused PDMS and the FN/protein layer. This thickens the oil layer further, creating an oil pool which is moved around the well, weakening the cell sheet-surface interactions and delaminating the cell sheet from the surface as it travels. This allows the FN/cellular layer to float freely on the oil interface, and makes it possible to transport the entire sheet from the substrate simply using filter paper (Fig. 1d). This mechanism is further supported by the observations that a FN sheet can be transferred directly without a cellular layer (Fig. 2a) and that some oil is transferred with the cells and FN (Fig. 3), suggesting the cells themselves are not an integral part of the removal mechanism. Moreover, the fact that we observed no difference in the release of fixed cells versus living cells, and that efficient sheet release was not possible on samples that were not fully infused, also confirms that the mechanism of removal relies primarily on the penetration of excess oil between the solid surface and the FN/cellular sheet, overcoming any attractive forces present between the two, and is not governed by the metabolic activity of the living cells and their interaction with the underlying substrate.

We found that initial removal of excess silicone oil and deposition of a FN layer were essential to allowing the cells to grow on infused polymers (Fig. 1). It is well known that FN is an abundant ECM protein with adhesion promoting properties^27^. Previous studies have shown that cells will not grow on PDMS alone^30^; we found similar results for our infused surfaces (Fig. 1a). However, once a FN layer was added to the surface growth, proliferation proceeded to a level equal to that found on TCPS controls (Fig. 1b). Previous work on infused polymers has shown that they naturally resist the adhesion of proteins^30^, adding to their value as anti-fouling materials. For this reason, the initial removal of the excess silicone oil with washing and blotting was necessary to permit the FN layer to weakly attach to the substrate and not wash away before the cell medium could be introduced.

While we did find that some silicone oil is transferred with the cell sheet upon removal (Fig. 3a,b), we did not see any signs of toxicity with the MSCs used here. In fact, the cell sheet was observed to grow right up to the base of the excess silicone droplets (Fig. 3b). These results are not surprising, as extensive investigations of silicones for implantation into the human body have shown no direct cytotoxic effects^29^. Moreover, our results show that the oil transferred with the cells can be washed away, resulting in a “healthy”, oil-free cell sheet construct. The fact that the oil droplets and even the oil layer coating the cells could be so easily removed may explain the lack of problems with growth and proliferation using this method.

The approach to cell sheet-release surfaces described here adds to the library of methods for producing intact, free-standing cell sheets or even individual cells. Using an immobilized liquid layer to create a surface with controllable adhesion based on the thickness of the layer is a completely different approach than previously developed technologies which rely on responsive polymers to change configuration upon input of a particular stimulus^9^,^10^,^11^,^17^,^18^. This may offer some important benefits, including application to cell types which are temperature, electro-, or photo-sensitive. Furthermore, the use of PDMS as a substrate permits easy benchtop fabrication of these materials with minimal training, and the relatively short time needed to release an entire sheet (5 ± 1.9 min) may permit more high-throughput applications than were previously possible with technologies that often required 40 min or more^14^. However, it should also be mentioned that our method, as currently described, does require physical movement of the excess oil around the plate, which if not done carefully can shred the cell sheet or result in the oil floating to the surface of the culture medium rather than remaining between the surface and the cellular layer. Future improvements could therefore involve a more automated approach to introducing and manipulating the excess oil to reduce or eliminate user error.

The use of infused PDMS for the growth and release of intact cell sheets also promises a number of future advantages in addition to the more immediate benefits described above. First, it is well known that MSCs can differentiate into different cell types ranging from neurons to osteoblasts, depending on the stiffness of the underlying substrate^5^. Furthermore, it has already been shown that the elastic modulus of PDMS can be easily tuned for the purpose of studying cell-surface mechanics^31^. We have shown that the tunability of the elastic modulus is preserved through the infusion process (Supplementary Fig. 2). Although the values measured here, ranging between 2.5 (±0.06) and 0.12 (±0.03) MPa (depending on the initial base:crosslinker ratio of the uncured polymer), are currently only suited to the growth and differentiation of osteoblasts, they may be expanded to include values required for adipose or muscle growth through the use of softer PDMS matrices. Second, the low cost of the silicone materials necessary to fabricate these surfaces (approximately $0.02/cm^2^) and the easy benchtop fabrication of the substrates may make this approach more appealing for high-volume or proof-of-principle tests where speed and low cost are considerations. Finally, the ability to mold PDMS substrate into arbitrary non-planar shapes may open doors to the use of more complex geometries or the incorporation of surface patterns to produce interesting 3D cell fabrics. Such benefits may serve to facilitate the further development and use of cell sheet engineering.

We believe that this work presents a novel alternative to current cell sheet-release technologies which may open the door to new applications of cell sheets in biotechnology and promote cell sheet-based therapies in medicine.

## Methods

### Preparation of infused polydimethylsiloxane (PDMS) well plates

Tissue culture polystyrene well plates were plasma-treated for 5 min at a power of 250 W and oxygen gas flow of 15 sccm in a plasma etching chamber (Plasma Etch, Inc. PE-200, Carson City, NV). PDMS was prepared with Sylgard 184 silicone elastomer (Dow Corning Corporation, Midland, MI). The base and curing agent were combined in a 10:1 ratio and mixed in a Thinky planetary centrifugal mixer (Thinky Corporation ARE-310, Tokyo, Japan) at 2000 rpm for 1 min, then again at 2200 rpm for 1 min. Well plates were removed from the plasma chamber and immediately filled with roughly 1 g of the PDMS mixture per well. The well plates were then placed inside a 70 °C oven (VWR Signature Forced Air Safety Oven 52201-216, Radnor, PA) for 5 min to ensure proper bonding between the plasma-treated polystyrene and PDMS. The PDMS-coated well plates were degassed in a vacuum chamber (VWR Symphony Vacuum Oven 414004-582, Radnor, PA) for a minimum of 1 hour and cured at 70 °C for at least 2 h. To infuse the PDMS coating, roughly 2 mL of Momentive 14 10A silicone oil (an excess amount) was placed in the well for 48 h, enough time for the PDMS to become completely infused.

### Removing Surface Oil and Fibronectin Deposition

After infusion, the excess silicone oil was removed from the surface to ensure deposition of a FN layer that is uniform and stable enough for the cells to attach and proliferate. First, the excess oil was aspirated out of the well plates and the surfaces were dried with a nitrogen gas gun. The sides of the wells were wiped with a lint-free tissue to remove any silicone oil residues. Silicone sponges (McMaster-Carr 86235K142 Robbinsville, NJ) were then placed inside the well plates for 2 h to absorb any surface silicone oil from the PDMS surface. Immediately before fibronectin deposition, the surfaces were again dried with a nitrogen gas gun and rinsed with 70% ethanol to sterilize.

Human plasma fibronectin (Millipore, FC0010, Billerica, MA) was used to coat the dried, infused PDMS well plates. PDMS well plates were incubated in 1.25 μg/mL, 2.5 μg/mL or 5.0 μg/mL solutions of fibronectin in Hank’s Balanced Salt Solution (HBSS) for 2 h at room temperature on a rocking platform. The positions of the plates on the platform were changed intermittently throughout the two hours. Well plates incubated in HBSS without any fibronectin acted as controls.

### Cell Culture

D1 ORL UVA [D1] (ATCC CRL1242, Manassas, VA) mouse bone marrow mesenchymal stem cells (MSCs) were used for all experiments. D1 cells were cultured in Dulbecco’s Modified Eagle Medium (DMEM), high glucose formulation with pyruvate (Gibco, 11995) with the addition of 10% Fetal Bovine Serum (Gibco, 10437) and 1% Penicillin-Streptomycin (Gibco, 15140). Cells were incubated at 37 °C with 5% CO_2_. For all experiments, cells were seeded at 5 × 10^4^/mL. For this study, cells in passages 26–39 were used.

### Cell Proliferation Assay

Cell proliferation was measured by staining the cells with Crystal Violet (CV) according to a modified version of a method developed by Gillies *et al.*^31^ The cells were washed twice with HBSS and then fixed with 1% glutaraldehyde (Alfa Aesar) in HBSS for 15 min at room temperature. The fixed cells were then washed with HBSS and stained with 0.1% CV in deionized, distilled (DI) water for 30 min. Well plates were rinsed with DI water at least 5 times to remove all residual CV. Well plates were photographed with Canon Rebel T4i (Canon). Image analysis was conducted with MATLAB to determine the density of the cell sheet in each well. Briefly, the images were first thresholded to determine the area of the cell sheet relative to the area of the well. Then the intensity values were used to generate a surface plot of the cell sheet area. Integrating under the surface plot gave the density of the cell sheet. This was normalized to a hypothetical cell sheet that was black (i.e. intensity value of 1) with the area equivalent to that of the well (i.e. 100% coverage). Thus a darker purple color gave a higher intensity value and hence a higher density value. One-way and two-way ANOVAs were conducted to determine significance among the various conditions. Statistics were carried out with IBM SPSS Statistics 22 (IBM).

### Detachment of Cell Sheets

Approximately 850 μL, or 90 μl/cm^2^, of filter-sterilized silicone oil (0.45 μm pore filter; needles: BD Precision glide 21 g (model 305165) or 23 g (model 305143); syringe: 1 mL NormJect Nonpyrogenic/nontoxic, 4010-200V0, Henke-Sass, Wolf, Tuttlingen, Germany), was injected under the cells onto the PDMS. Care must be taken not to puncture through the PDMS layer, otherwise delamination of PDMS will begin (delamination from punctures is not immediately noticeable as it occurs over the course of 2–3 days). The silicone oil formed a pool underneath the media solution on the surface of the polymer. This bubble was slowly rolled around to peel off the cell sheet, which rested on the oil-media interface. In places where the cells were more strongly adhered, flat-tipped tweezers were used to guide the silicone oil under the cell sheet to lift it off.

The cell sheet was transferred to a new culture surface for further culturing and processing using filter paper. Briefly, the silicone oil bubble was aspirated out, and filter paper circle (Whatman 1001-329, Buckinghamshire, UK) was pressed into the well over top of the cell sheet. Media was aspirated out from above the filter paper so that the cell sheets would adhere to the filter paper through capillary force. The filter paper was then peeled off and transferred to the new culture surface (35 mm petri dish) where fresh media was added to detach the cell sheet from the filter paper. The cell sheet detached from the filter paper in less than 5 min, at which point it could be removed from the media. The new culture surface with the transferred cells was then placed into the incubator to allow the cells to continue growing and form strong attachment bonds to the new surface. To ensure that the cells had reattached sufficiently, at least 1 h was allowed for the cells to reattach before further processing was done.

### Cell Sheet Viability

To determine the viability of the cell sheets after transfer, Calcein AM was used to detect intracellular esterase activity and Sytox Orange Nucleic Acid stain to detect membrane integrity. Transferred cells were washed with HBSS twice and then incubated in 2uM Calcein AM (Molecular Probes, C3099) and 2uM Sytox Orange (Molecular Probes, S11368) in Serum-free DMEM at 37 °C for 1 h. The stained cells were then washed twice with HBSS and imaged in Live Cell Imaging Buffer (Molecular Probes, A1429DJ). Cells growing on infused PDMS (state of cells before detachment) and cells growing on TCPS were also stained and imaged as controls. A Zeiss LSM 710 confocal microscope was used to image the cells using the preloaded excitation and emission wavelengths set for the Calcein AM and Sytox Orange dyes. Image analysis (n = 3) was used to calculate the coverage of the two dyes relative to the total dyed cell sheet area to quantify the viability. Image analysis was carried out with Zen image acquisition software (Zeiss) and MATLAB (Mathworks).

Experiments using dyed silicone oil to determine the localization of the oil after transfer were performed using a 1:1 mixture of silicone oil and difluoro{2-[1-(3,5-dimethyl-2*H*-pyrrol-2-ylidene-*N*) ethyl]-3,5-dimethyl-1*H*-pyrrolato-*N*}boron dye (Sigma-Aldrich). For these experiments we used small groups of individual cells, as opposed to an entire sheet, to ensure the maximum possible contact of the oil with the cells and thus the maximum possible coating or uptake of the oil by the cells. Thus these cells were not as securely attached to the surface and were more easily washed away. To ensure that any apparent removal of the dye was due to the washing rather than bleaching of the fluorophores, cells were released from the surface and immediately imaged, then washed and imaged again.

### Immunofluorescent staining

Cell sheets were washed with HBSS twice and then fixed with 1% glutaraldehyde in HBSS for 20 min at 4 °C. Fixed cells were then washed with HBSS and permeabilized with 0.5% Triton X-100 in HBSS for 10 min and subsequently blocked with stain buffer containing 0.2% BSA in PBS (Pharmingen) for 30 min at room temperature. Cells were then immunostained with a 1:200 dilution (1 μg/ml) of rabbit polyclonal anti-fibronectin antibody (SantaCruz Biotechnology, sc-9068). Cell sheets were washed with BSA buffer twice before reacting with a 1:2000 dilution (1 μg/ml) of Alexa Fluor 633 conjugated goat anti-rabbit secondary antibody for 1 h at 4 °C. Cells were also co-stained with a 1:1500 dilution of Alexa Fluor 546 conjugated phalloidin to stain f-actin and 1 μg/ml Hoechst 33342 nuclear stain. Cells were washed twice in BSA buffer before imaging. Cell sheets before detachment and confluent cell layers grown on TCPS coated with fibronectin were also immunostained and imaged as controls. Cells were imaged with a Zeiss LSM 710 confocal microscope using the preloaded excitation and emission wavelengths set for Alexa Fluor 633, Alexa Fluor 546 and Hoechst dyes. A confocal z-stack of the cells was taken in each well (n = 3). The base of the cell sheet (i.e. the imaging plane that showed the basal layer of the cell sheet in focus) was used for image analysis to calculate the amount of fibronectin per nuclei and f-actin per nuclei to quantify ECM and cell spreading morphologies. Image analysis was carried out with Zen image acquisition software (Zeiss) and MATLAB (Mathworks).

### Preparation of PDMS substrates with variable elastic modulus

PDMS (Dow Sylgard 184 polydimethylsiloxane) samples for elastic modulus testing were made by combining polymer base to curing agent at 5:1, 10:1, 20:1 and 30:1 ratios. After curing, the samples were immersed in 10 cSt silicone oil for a minimum of one week to allow for maximum absorption. Control samples were not placed in silicone oil. The samples were placed between silicone sponges for approximately 10 min to remove any excess surface oil. The Young’s moduli of the samples were determined using the Agilent Nano Indenter G200 with a (100 μm diameter) diamond flat punch. During each test, the flat punch contacted the surface of the sample and oscillated to determine the complex moduli of the samples. The complex modulus was determined at four points on each sample. Young’s modulus was then calculated using a Poisson’s ratio of 0.5.

### Visualization of the replenishment of the oil overlayer

The surface of the infused PDMS samples over time after removal of the excess oil with silicone sponges were imaged using phase-contrast optical microscope (AX10, Carl Zeiss AG, Oberkochen, Germany) at low magnification. Silicone sponges were placed in contact with the sample surfaces for 24 h, then removed and immediately imaged for a t = 0 measurement. Images were taken every hour thereafter for up to 48 hours.

## Additional Information

**How to cite this article**: Juthani, N. *et al.* Infused polymers for cell sheet release. *Sci. Rep.* **6**, 26109; doi: 10.1038/srep26109 (2016).

## Supplementary Material

Supplementary Information

Supplementary Video 1

Supplementary Video 2
