# Supplementary material for: Infused polymers for cell sheet release
Source: Sci Rep. 2016 May 18;6:26109. doi: 10.1038/srep26109 (PMC4870626; doi:10.1038/srep26109)
Supplement: Supplementary Information [file srep26109-s1.pdf]

Supplementary Information

Infused polymers for cell sheet release

Nidhi Juthani,[1,2] Caitlin Howell,[1,2,3] Haylea Ledoux,[1] Irini Sotiri,[1] Susan Kelso, [1,2] Yevgen Kovalenko,[1,2] Amanda Tajik,[1,2] Thy L. Vu,[1] Jennifer J. Lin,[1,2] Amy Sutton,[4] and Joanna Aizenberg[1,2,4,5]

[1]Wyss Institute for Biologically Inspired Engineering, 60 Oxford Street, Cambridge, Massachusetts 02138, United States. [2]John A. Paulson School of Engineering and Applied Sciences, Harvard University, 29 Oxford Street, Cambridge, Massachusetts 02138, United States. [3]Department of Chemical and Biological Engineering, University of Maine. 5737 Jenness Hall, Orono, ME 04469, United States. [4]Department of Chemistry and Chemical Biology and [5]Kavli Institute for Bionano Science and Technology, Harvard University, 12 Oxford Street, Cambridge, Massachusetts 02138, United States. Correspondence should be addressed to C.H. (caitlin.howell@maine.edu) or J. A. (jaiz@seas.harvard.edu)

Content

Supplementary Figures

Supplementary Figure 1: Swelling curve for a 2 mm-thick PDMS layer
Supplementary Figure 2: Elastic moduli of infused PDMS made with different cross-linker ratios
Supplementary Figure 3: Optimization of the fibronectin concentration for cell sheet growth.
Supplementary Figure 4: Replenishment of the silicone oil overlayer over time.
Supplementary Figure 5: Images of a cell sheet before and after transfer.

Supplementary Online Resources

Supplementary Video 1: Detachment of cell sheet with excess silicone oil
Supplementary Video 2: Transfer of cell sheet to a new surface using filter paper

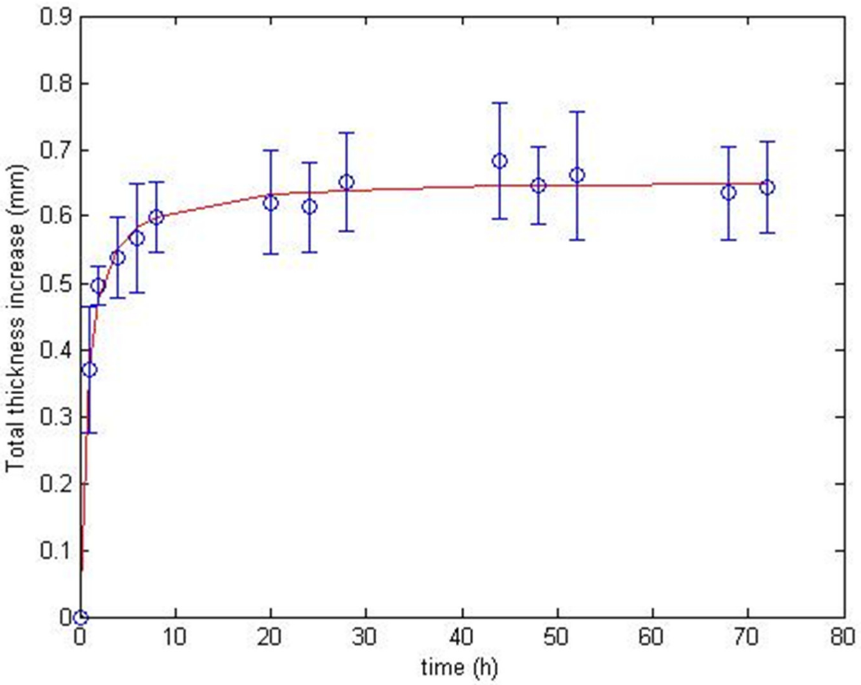

Supplementary Figure 1: Thickness versus time for a 2 mm-thick PDMS slab undergoing infusing with 10 cSt silicone oil. The thickness. Complete infusion, as determined by the time to equilibrium thickness, occurs after approximately 20 h of exposure to excess silicone oil.

To ensure that complete infusion of the PDMS layer had occurred after 48 hours, the change in thickness of a 2 mm-thick slab of PDMS undergoing infusion was measured over time. The thickness was chosen to be twice as thick as the PDMS coating of the wells of the plate used to grow the cells (~1 mm) to account for the fact that the slabs were infused from both the top and the bottom, rather than just the top (as would be the case for the coating in the well plate). The slabs were immersed in excess 10cSt silicone oil, and removed and photographed from the side periodically to determine thickness change. It was found through fitting with a modified Langmuir isotherm function that the thickness of the slab stopped increasing after 21.7 h of exposure to the excess silicone oil.

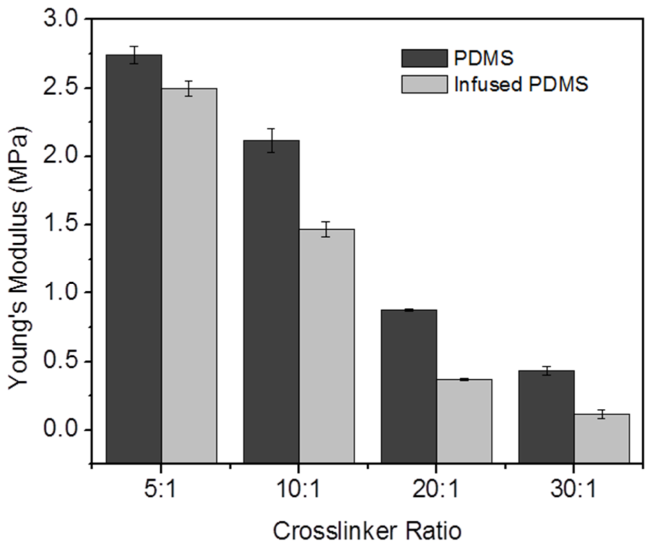

Supplementary Figure 2: Elastic moduli of infused PDMS made with different cross-linker ratios.

The values for the elastic moduli as determined by the indentation method were 2.74 (±0.07) MPa, 2.12 (±0.09) MPa, 0.89 (±0.01) MPa and 0.43 (±0.03) MPa for the 5:1, 10:1, 20:1 and 30:1 controls, respectively. Young’s moduli for the 5:1, 10:1, 20:1 and 30:1 infused PDMS samples were 2.50 (±0.06) MPa, 1.47 (±0.05) MPa, 0.37 (±0.01) MPa and 0.12 (±0.03) MPa, respectively. All cross-linker ratios demonstrated a decrease in Young's modulus following infusion with 10cSt silicone oil. 5:1 samples were found to have the smallest decrease in elastic moduli with an average difference of 0.24 MPa between its non-infused and infused state. 10:1, 20:1 and 30:1 samples had an average decrease in elastic moduli of 0.65 MPa, 0.52 MPa and 0.31 MPa, respectively.

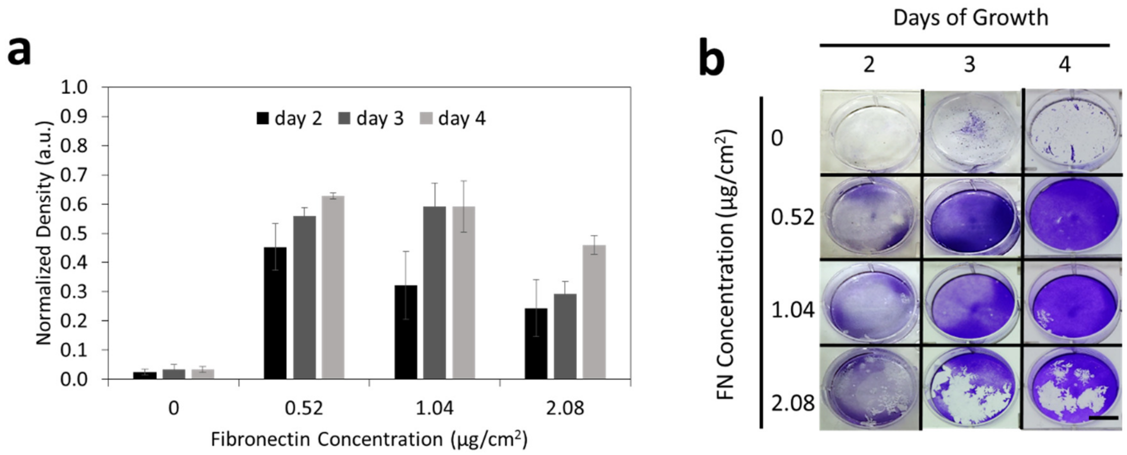

Supplementary Figure 3: Optimization of the fibronectin (FN) concentration for cell sheet growth. (a) Normalized density of cells grown on infused PDMS with increasing concentration of FN coating the wells for 2, 3, and 4 days. The density was found to be highest after 4 days for wells coated with either 0.52 or 1.04 µg/cm^2. (b) Images of the surfaces used for analysis. The cells are stained with CV for easier visualization. Scale bar, 1cm.

To test the effect of fibronectin concentration on cell sheet growth in this system, cells were grown on infused PDMS surfaces following incubation in 0, 1.25, 2.5 or 5.0 µg/mL fibronectin solutions (0, 0.52, 1.04, or 2.08 µg/cm^2) for 2, 3 or 4 days. After the specified growth time, cells were fixed, stained, photographed and peeled off for further processing. As expected, wells with no fibronectin showed very little cell proliferation. Interestingly, wells incubated in 2.08 µg/cm^2 fibronectin also had significantly less cell proliferation than 0.52 µg/cm^2 and 1.04 µg/cm^2 across all 3 days ($P < 0.05$). This may be attributed to clumping and aggregation of the fibronectin protein, preventing the RGD binding site from being exposed to the cells.[26] As there was no significant difference in cell proliferation between wells incubated in 0.52 µg/cm^2 and 1.04 µg/cm^2 fibronectin ($P = 0.567$), 0.52 µg/cm^2 fibronectin solutions were used for all other experiments.

Optimization of the incubation time for cell sheet growth in this system was also investigated. Each of the incubation times tested (2, 3, and 4 days) had its own advantages and disadvantages and incubation time selection was dependent on the degree of confluence that was desired. Cell sheets harvested after 2 days growth readily detached with just the advancement of the silicone oil interface, but produced less confluent sheets that required more careful handling. Cell sheets harvested after 4 days growth required the use of tweezers to aid in detachment but produced much more robust confluent sheets that were easy to handle and transfer. For cell sheets harvested after 3 days of growth, some areas detached with the advancement of the interface, whereas others needed the help of tweezers. These sheets were much more robust and easier to handle compared to those harvested after only 2 days growth. In all cases, cell sheets could be released from the surface within minutes, with an average release time of ~3.5 min.

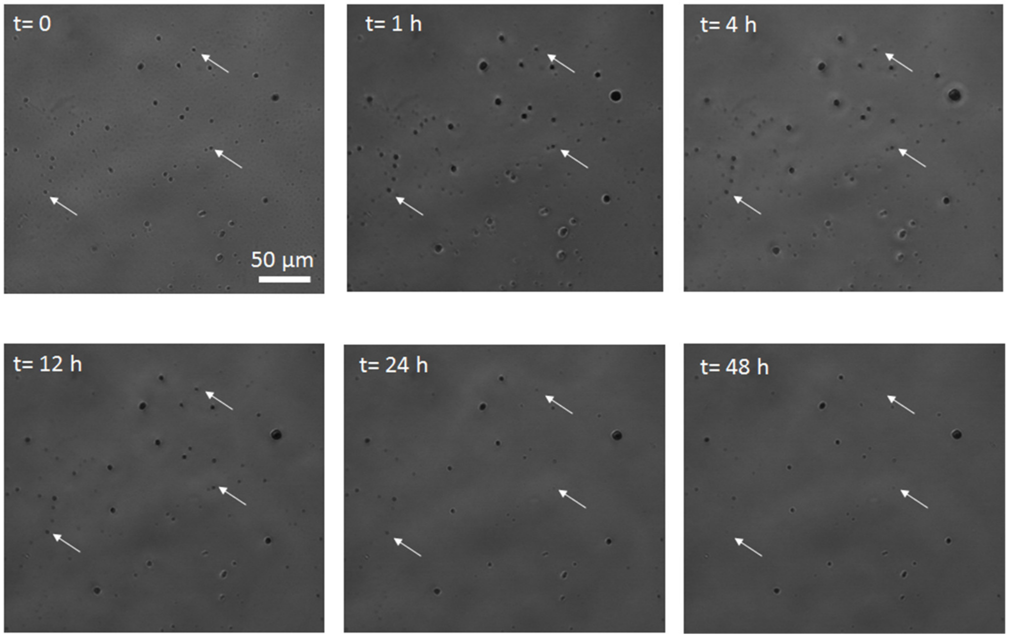

Supplementary Figure 4: Replenishment of the silicone overlayer over time after removal via absorption by silicone sponges. The oil layer is initially depleted upon removal of the sponge, then begins to replenish at 3 h, and continues over the entire 48 h growth window of the cells. Arrows indicate defects which slowly disappear due to filling with oil. Other larger defects initially appear to become larger due to the deformation of the oil layer surrounding them, but then also decrease in size as they are slowly filled in.

An infused PDMS surface was visualized over time via brightfield microscopy after removing the excess oil with silicone sponges. The images showed a slow replenishment of the oil on the surface over 48 hours, as indicated by the gradual disappearance of surface defects. This supported the hypothesis that the FN/cellular layer was sitting upon an immobilized liquid layer which, upon addition of excess oil, thickened and released the sheet from the surface.

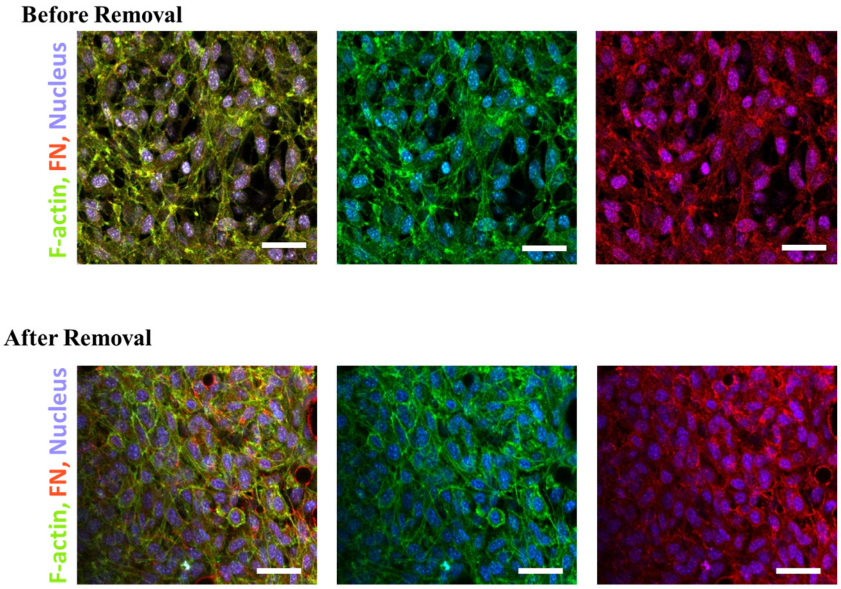

Supplementary Figure 5: Images of a cell sheet before and after transfer. Scale bar, 50μm. Staining was performed to ensure that no morphological changes were occurring during the cell sheet transfer. Confocal images of cell sheets grown on infused PDMS were taken before removal and after removal. F-actin appears green, fibronectin – red, and the nuclei – blue. All three components were found to be present both before and after removal, and the organization and morphology of the sheet overall appeared unchanged. Furthermore, the fibronectin layer can clearly be seen underneath the cells after removal, in agreement with the results from **Fig. 2a.**
